# Artificial intelligence algorithm to predict the need for critical care in prehospital emergency medical services

**DOI:** 10.1186/s13049-020-0713-4

**Published:** 2020-03-04

**Authors:** Da-Young Kang, Kyung-Jae Cho, Oyeon Kwon, Joon-myoung Kwon, Ki-Hyun Jeon, Hyunho Park, Yeha Lee, Jinsik Park, Byung-Hee Oh

**Affiliations:** 1 Artificial Intelligence and Big Data Research Center, Sejong Medical Research Institute, 20, Gyeyangmunhwa-ro, Gyeyang-gu, Incheon, Republic of Korea; 2 VUNO, Seoul, South Korea; 3 Department of Emergency Medicine, Mediplex Sejong Hospital, 20, Gyeyangmunhwa-ro, Gyeyang-gu, Incheon, Republic of Korea; 4 Division of Cardiology, Cardiovascular Center, Mediplex Sejong Hospital, Incheon, South Korea

**Keywords:** Emergency medical service, Triage, Artificial intelligence, Deep learning

## Abstract

**Background:**

In emergency medical services (EMSs), accurately predicting the severity of a patient’s medical condition is important for the early identification of those who are vulnerable and at high-risk. In this study, we developed and validated an artificial intelligence (AI) algorithm based on deep learning to predict the need for critical care during EMS.

**Methods:**

We conducted a retrospective observation cohort study. The algorithm was established using development data from the Korean national emergency department information system, which were collected during visits in real time from 151 emergency departments (EDs). We validated the algorithm using EMS run sheets from two EDs. The study subjects comprised adult patients who visited EDs. The endpoint was critical care, and we used age, sex, chief complaint, symptom onset to arrival time, trauma, and initial vital signs as the predicted variables.

**Results:**

The number of patients in the development data was 8,981,181, and the validation data comprised 2604 EMS run sheets from two hospitals. The area under the receiver operating characteristic curve of the algorithm to predict the critical care was 0.867 (95% confidence interval, [0.864–0.871]). This result outperformed the Emergency Severity Index (0.839 [0.831–0.846]), Korean Triage and Acuity System (0.824 [0.815–0.832]), National Early Warning Score (0.741 [0.734–0.748]), and Modified Early Warning Score (0.696 [0.691–0.699]).

**Conclusions:**

The AI algorithm accurately predicted the need for the critical care of patients using information during EMS and outperformed the conventional triage tools and early warning scores.

## Introduction

An important objective of emergency medical services (EMSs) is to provide appropriate prehospital management and transfer to the relevant emergency department (ED) based on a patient’s status [1]. Several prognosis prediction tools have been developed for EMS but are limited to specific situations, such as trauma [2]. Although some efforts have been made to apply existing ED triage tools and early warning scores to EMSs, these tools have so far performed unsatisfactorily [3].

In EMS, accurately predicting the need for critical care is important for the early identification of the vulnerability and high-risk of patients, and for deciding the most appropriate management during transfer [4]. If the patient is expected to require critical care, the EMS technician must pass through the nearest low-level ED to a high-level ED [5]. Accurate tools for predicting prognosis are important for communication between the prehospital EMS technician and hospital medical staff to provide online medical directions and prepare in-hospital management [6, 7].

The goal of this study was to develop and validate an artificial intelligence (AI) algorithm based on deep learning to predict the need for critical care of patients in EMSs accurately. Deep learning could overcome the limitations of conventional statistical methods and has recently achieved state-of-the-art performance in several domains, including medical imaging and outcome prediction [8–10]. To the best of our knowledge, this study is the first to predict severity in EMS using an AI algorithm.

## Methods

### Study design and setting

This was a multicenter retrospective cohort study, not a blind study. Furthermore, the study was entirely separated between development and external validation data. To establish the AI algorithm, we used the Korean national emergency department information system (NEDIS), which collects all patient visits in real time from 151 EDs in Korea. To externally validate our model, EMS run sheets from patients who visited two EDs were used. Specifically, the EMS run sheets contain information on when patients were contacted by an EMS. The run sheets were saved as electronic medical records. The sample size of the validation dataset was determined using an accurate algorithm in a previous study [11].

The data comprised age, sex, chief complaint, time from symptom onset to visit (or EMS contact), trauma, initial vital signs (systolic blood pressure, diastolic blood pressure, heart rate, respiratory rate, and body temperature), and mental status; these data were used as the predictor variables. The endpoint of this study was critical care (admission to intensive care unit). For the stabilized training, the input variables were normalized with a z-score.

The institutional review boards of Sejong General Hospital (2019–0212) and Mediplex Sejong Hospital (2019–049) approved this study protocol and waived the need for informed consent because of the impracticality and minimal harm involved.

### Selection of participants

**Fig. 1:**
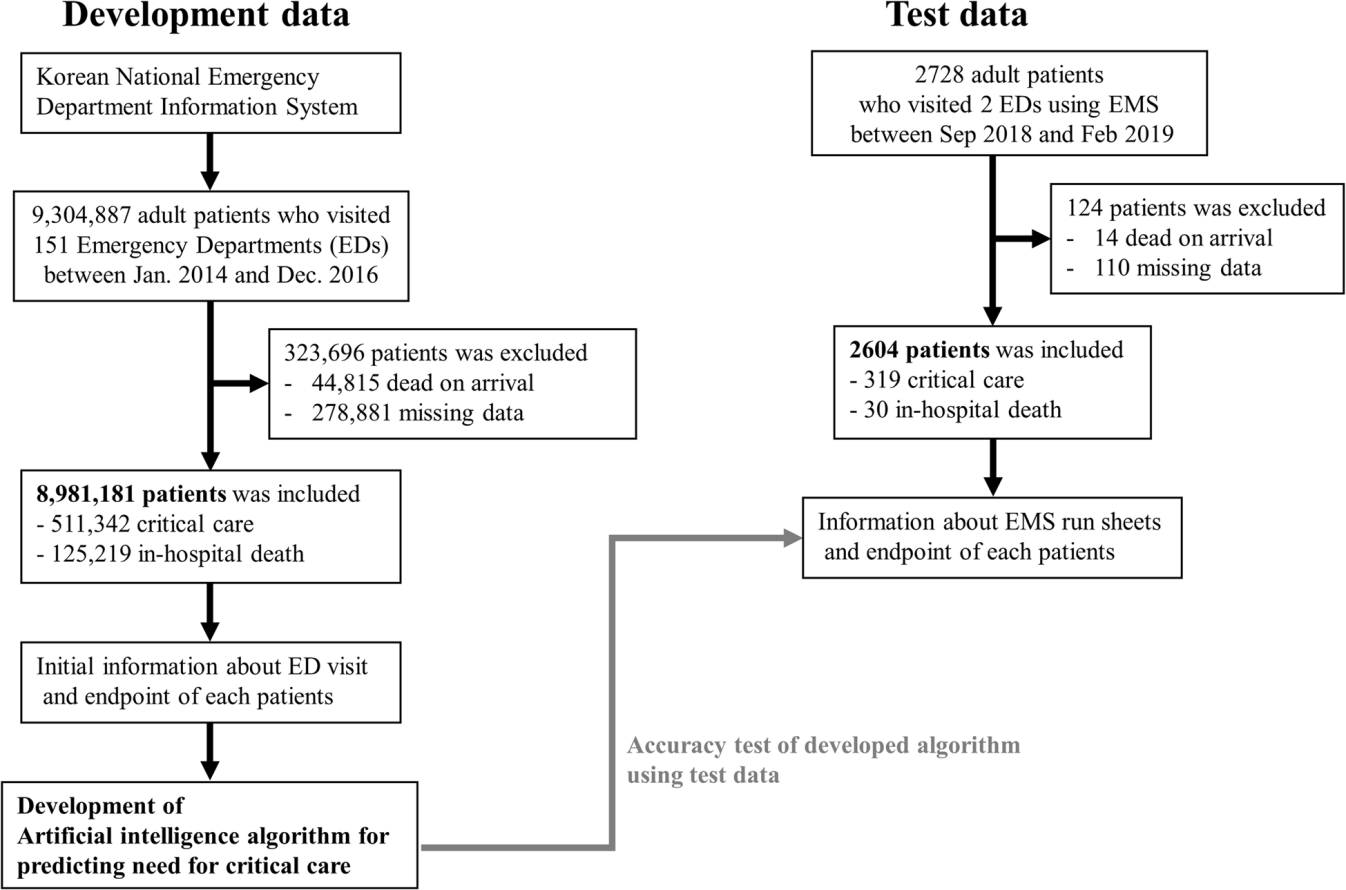
Study flowchart. Legends: ED: emergency department; EMS: emergency medical service

The study participants were adult patients (aged ≥18 years) who visited EDs. From the development data (NEDIS), we selected adult patients who visited EDs between January 2014 and December 2016. Moreover, we selected patients who visited two EDs using EMSs between September 2018 and February 2019 as the test data. We excluded subjects who were declared dead on arrival and those for whom data were missing, as shown in Fig. 1.

### Development of AI algorithm based on deep learning

To establish our algorithm, development data (NEDIS) were utilized. To classify the presence of critical care needs, we used feedforward networks (5 hidden layers, 89 nodes, and batch normalization [12, 13]), which train the output using the softmax classifier. We applied a dropout rate of 0.5 at each layer for regularization and a rectified linear unit was used for the activation function. The Adam optimizer was used to improve the efficiency of optimization, while the cross-entropy loss function was used to minimize the prediction loss based on a supervised learning. In addition, we used *TensorFlow* (the Google Brain Team, Mountain View, United States) as the backend [14]. The calibration plot and Brier score are described in a supplemental figure.

### Performance test of AI algorithm and comparison with conventional methods

We compared the performance of the algorithm in terms of predicting critical care with those of the Emergency Severity Index (ESI), Korean Triage and Acuity System (KTAS), Modified Early Warning Score (MEWS), and National Early Warning Score (NEWS). The ESI is a globally used five-level ED triage algorithm, initially developed in 1999 [15, 16]. It is based on the severity of patients’ healthcare problems and the number of resources that is anticipated to require. KTAS was developed in 2012 based on the Canadian Triage and Acuity Scale and has been used nationwide as a triage since 2016 in Korea [11, 17, 18]. KTAS is a five-level ED triage algorithm that considers symptoms, pain, and physiological values. Three medical staff members with more than 5 years of experience in clinical practice in an ED participated in this study. They decided the ESI and KTAS levels with information from the EMS run sheets for patients in the test data. Conflicting results were decided by discussion.

MEWS is a widely used tool for predicting severity and deterioration, and is calculated using systolic blood pressure, heart rate, respiratory rate, body temperature, and mental status [19]. NEWS was developed in the United Kingdom. It is a popular aggregated scoring system that considers respiratory rate, oxygen saturation, temperature, systolic blood pressure, heart rate, and mental status [20]. The MEWS and NEWS scores have been well-validated and used globally. In previous studies, some efforts have been made to apply these early warning scores to EMSs [21]. We calculated the MEWS and NEWS scores based on information from the EMS run sheets. The EMS run sheets comprise data at the time of first contact of EMSs with each patient.

We validated the developed algorithm using exclusively divided test data. The performance measures were taken as the area under the receiver operating characteristic curve (AUC), sensitivity, specificity, positive predictive value (PPV), negative predictive value (NPV), and F1 score. The AUC is a frequently used metric and shows the sensitivity against 1-specificity [22]. Based on previous studies, we used levels 1–2, levels 1–2, points 3–14, and points 5–20 to predict the critical care with the ESI, KTAS, MEWS, and NEWS, respectively [11, 17–21]. When evaluating the continuous score predicted by the AI algorithm, we fixed the sensitivity as 0.8. Furthermore, we evaluated the 95% confidence interval using bootstrapping (10,000 times resampling with replacement) [23]. We used the *ROCR* package in R (R Development Core Team, Vienna, Austria) for these analyses.

### Combining the AI algorithm and conventional triage tools

With the aim of developing a high-performance algorithm, we combined the AI algorithm with conventional triage tools. This method is called *ensemble* [24]. A major limitation of the ESI and KTAS, as reported by previous studies, is the decreasing accuracy attributed to patients at mid-level, such as level 3. We applied the AI algorithm for patients at level 3 for each ESI and KTAS, and validated the performance of the two ensemble models (AI+ESI and AI+KTAS). For this, patients at levels 1 and 2 were predicted to be critical while patients at levels 4 and 5 were predicted to be noncritical. The AI algorithm only evaluated the patients at level 3.

## Results

In the development data, in total, 9,304,887 ED visits to 151 hospitals were included in the NEDIS. We excluded 323,696 visits because of the exclusion criteria: 44,815 were declared dead on arrival, while data were missing for 278,881 visits. No significant differences in the predictor variables were observed between the included and excluded study subjects due to the missing variables. Thus, the study subjects included 8,981,181 ED patients; 511,342 ended up in critical care (5.7%) and 125,219 (1.4%) died in hospital.

In the case of the test data, after excluding 124 patients (14 dead on arrival and 110 missing data), validation of the AI algorithm in EMSs was performed using 2604 patients from two hospitals, whose endpoints were 319 in critical care (12.3%) and 30 of whom died in hospital (1.2%).

The baseline characteristics of the development and test data are shown in Table 1. These two data were exclusively divided, and their characteristics are significantly different.

**Table 1:** Baseline characteristics^a^

Characteristics	Development data (***n*** = 8,981,181)	Test data (***n*** = 2604)	*p*-value^b^
**Data**			
Data type	National Emergency Department Infromation System (NEDIS)	Emergency Medical Service (EMS) Run Sheets	
Data source	Emergency department visit data	EMS run sheets	
Data period	1 January 2014–30 June 2016	1 September 2018–28 February 2019	
**Age**	49.9 ± 18.9	61.5 ± 18.6	< 0.001
**Female, No.(%)**	4,511,654 (50.2%)	1411 (54.2%)	< 0.001
**Initial vital signs, mean ± SD**			
Systolic BP (mmHg)	131.2 ± 23.3	132.0 ± 24.6	0.271
Diastolic BP (mmHg)	79.3 ± 13.9	83.7 ± 17.4	< 0.001
Heart rate (/min)	83.8 ± 16.2	85.5 ± 20.5	< 0.001
Respiratory rate (/min)	19.6 ± 2.7	17.7 ± 3.3	< 0.001
Body temperature (°C)	36.7 ± 0.7	36.7 ± 0.8	< 0.001
**Mental status, No.(%)**			< 0.001
Alert	8,674,058 (96.6%)	2513 (96.5%)	
Reacting to voice	161,624 (1.8%)	20 (0.8%)	
Reacting to pain	113,192 (1.3%)	46 (1.8%)	
Unresponsive	32,310 (0.3%)	25 (1.0%)	
Trauma, No.(%)	2,536,815 (28.2%)	550 (21.1%)	< 0.001
**Symptome onset to visit (contact) time, No.(%)**			< 0.001
–24 h	5,394,527 (60.1%)	2105 (80.8%)	
24 h–72 h	2,666,179 (29.7%)	448 (17.2%)	
72 h–7 Days	536,525 (6.0%)	38 (1.5%)	
7 Days–30 Days	258,641 (2.9%)	12 (0.5%)	
30 Days–	125,312 (1.4%)	1 (0.0%)	
**Outcomes, No.(%)**			
Critical care	511,342 (5.7%)	319 (12.3%)	0.006
In-hospital mortality	125,219 (1.4%)	30 (1.2%)	< 0.001
Hospitalization	2,443,994 (27.1%)	1003 (38.5%)	< 0.001

^a^BP denotes blood pressure

^b^The alternative hypothesis for this *p*-value was that there is a difference between the development and test data group for each variable

**Fig. 2:**
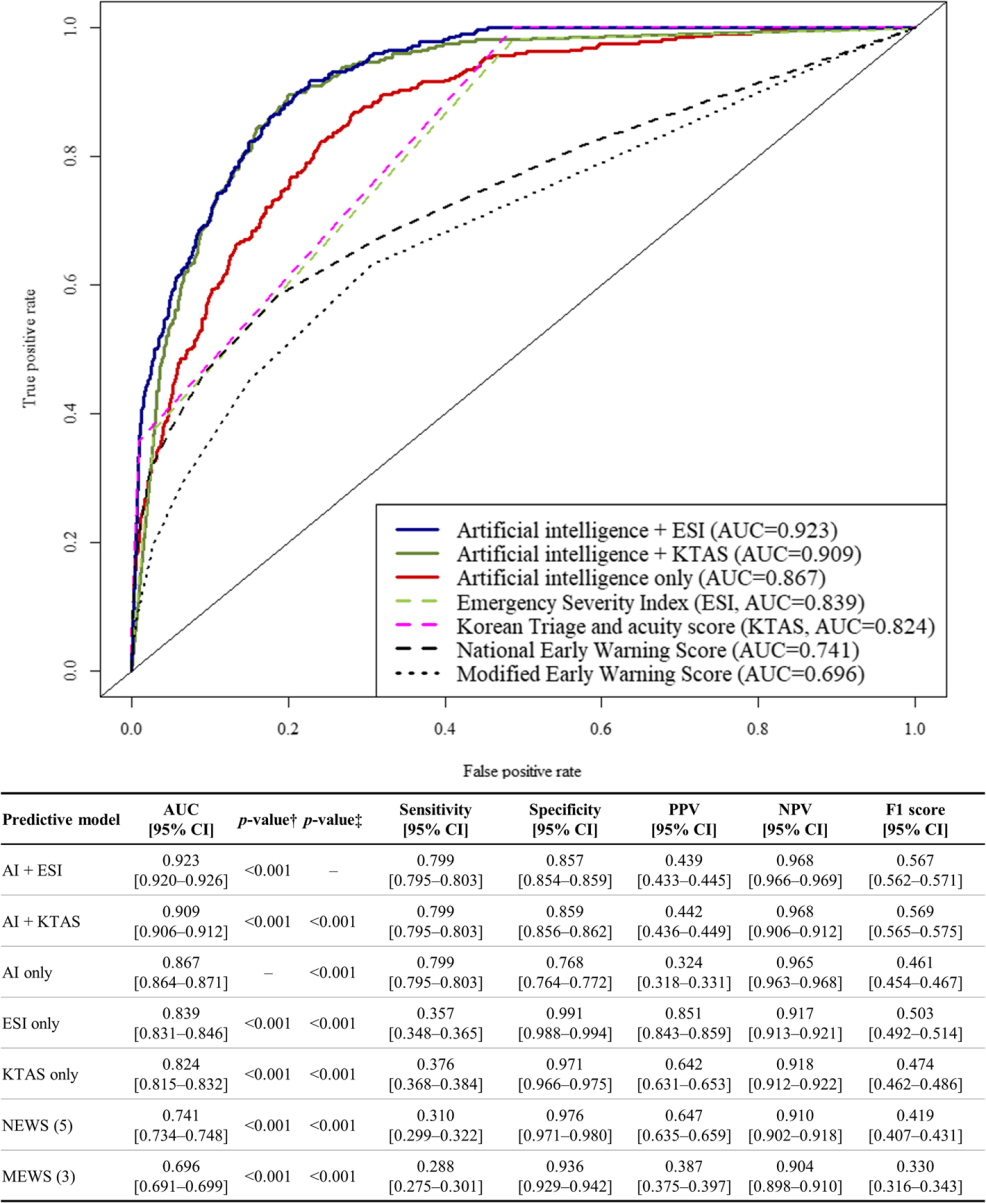
Receiver operating characteristics curve for predicting critical care Legends: *AI: artificial intelligence; AUC: area under the receiver operating characteristics curve; CI: confidence interval; ESI: Emergency Severity Index, KTAS: Korean triage and acuity system; MEWS: modified early warning score; NEWS: national early warning score; NPV: negative predictive value; PPV: positive predictive value. †The alternative hypothesis for this *p*-value was that there is a difference between the artificial intelligence algorithm and the other predictive methods. ‡The alternative hypothesis for this *p*-value was that there is a difference between the ensemble model, combining artificial intelligence and the ESI, and the other predictive methods

As shown in Fig. 2, the AUC of the AI algorithm was 0.867 (95% confidence interval [0.864–0.871]), which outperformed the ESI (0.839), KTAS (0.824), NEWS (0.741), and MEWS (0.696). Furthermore, the ensemble algorithm AI + ESI (0.923 [0.920–0.926]) significantly outperformed the other ensemble algorithm AI + KTAS (0.909 [0.906–0.912]), AI algorithm, and other conventional methods.

## Discussion

This study demonstrated that the AI algorithm accurately predicted the need for critical care in a prehospital EMS situation.

Predicting the need for critical care is important for selecting the destination ED and for providing the appropriate management during transfer [4, 5]. In addition, tools for accurately predicting the prognosis and treatment are important to communicate between the prehospital EMS technician and hospital medical staff [6, 7]. However, most triage tools in prehospital situations were developed for trauma patients only, and there is no generalized tool that covers all EMS situations [2, 25]. Although the conventional triage tools of EDs have been applied to predict the need for critical care at prehospital situations [21, 26, 27], they showed an unsatisfactory performance in predicting prognosis.

The important finding of this study is that the predictive performance of the AI model based on deep learning is superior to those of the conventional triage tools and scoring systems. In addition, three ED medical staff members were involved in deciding the level of triage with EMS run sheets. Interestingly, the accuracy of the AI algorithm was better than the accuracy of the decision of the expert medical staff. The AI algorithm performs automatic calculations based on basic information and does not require expert judgment and medical experience.

Deep learning can obtain a high performance without prior knowledge to train the model; thus, indicating that deep learning somehow automatically learns the feature relationship among input variables. In our previous study, we developed an AI algorithm based on deep learning for predicting the critical care of patients in an ED [11]. From the previous study, we found that conventional statistical methods such as logistic regression may have difficulty in determining the relationship among input variables [10, 28, 29]. As a large number of input variables were utilized, the dimensionality of the input increased. This somehow indicates that the process of feature extraction by humans should be required and effort should be made to determine the relationship between input variables.

Meanwhile, deep learning includes feature learning, which allows the model to automatically learn the relationships and characteristics between input variables required to perform a task [30]. As shown in our previous studies, deep learning could be used to understand the connection between features and outperformed conventional and other machine learning methods [9, 11, 31]. It is important to note that feature learning is not designed by humans in deep learning. As this process evolves automatically, it will be easier and more effective to identify intricate structures in high-dimensional data without information loss, and will result in end-to-end learning, which requires little engineering by humans. Finally, it can be easily and quickly applied to other tasks [30].

In addition, one of the well-known concepts in the use of deep learning is the importance of the amount of data. The accumulation of numerous data for decades advanced the performance improvement of deep learning. Likewise, the performance of the model based on deep learning depends greatly on the amount of data. In this study, we used the NEDIS data, which comprise millions of data. We believe that this amount of accumulated data would be more suitable for deep learning than other approaches. Moreover, we only used the initial vital signs for patients (assuming that it would be difficult to measure vital signs several times during transport). We considered the simple DNN model as more suitable than LSTM. Because the LSTM is based on sequential information.

The prevention of overfitting into a single hospital is an important issue. Further, it is crucial to verify whether the model was overfitted to a specific environment. Thus, the acquisition of external validation data is important. Wolpert explains this in the “No Free Lunch” theorem: If optimized in one situation, an algorithm cannot produce good results in other situations [32]. In this study, the development (ED) and test data (EMS) were exclusively divided. More specifically, the model was evaluated on the external dataset and could possibly avoid overfitting in one environment.

**Fig. 3:**
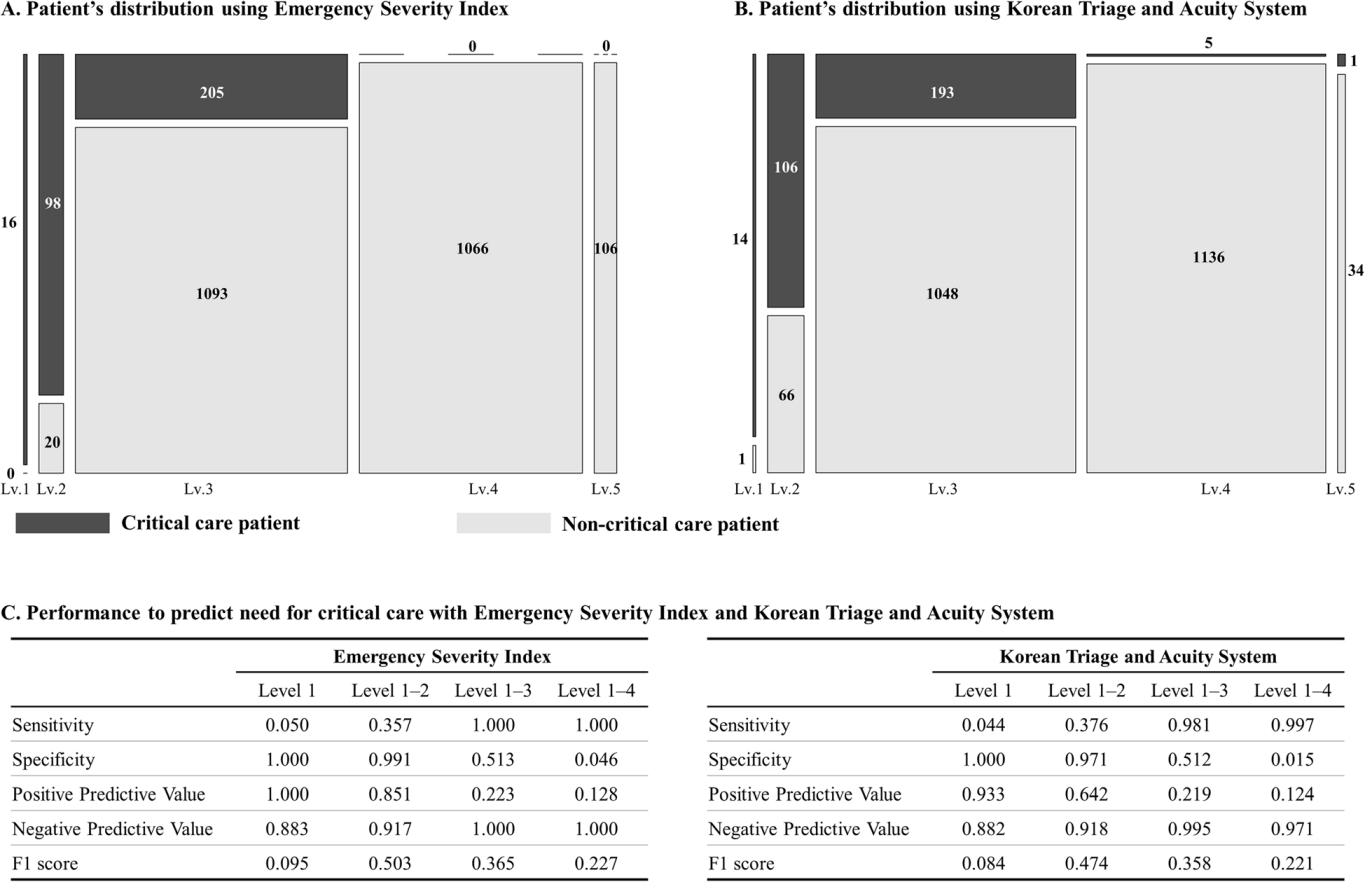
Patient distributions and performance using conventional triage systems

A major limitation of conventional triage tools is their low accuracy at the middle level [18, 33, 34]. As shown in Fig. 3, at level 3 triages, the population were mixed as critical care and non-critical care patients. If the patients at level 3 can be distinguished, we consider that the accuracy of predicting the outcome will increase. Therefore, we made effort to apply the algorithm for patients at level 3.

In this study, we developed the ensemble algorithm to evaluate level 3 patients and confirmed a higher performance with the AI+ESI algorithm. It is interesting to note that the combination of the expert opinion (ESI level) and the AI algorithm exhibits a more accurate performance. These results provide an opportunity to solve the problem for researchers in other medical fields. For example, in previous studies of urology, AI algorithms have been applied for the prediction of prostate biopsy results and the recurrence-free probability of bladder cancer [35]. In addition, the in-hospital and long-term mortalities of patients with cardiovascular diseases have been predicted using AI algorithms in several previous studies.

Our study has several limitations. First, deep learning is considered a black box. Although we can fit the AI algorithm based on deep learning, it is difficult to fully understand how the model predicts critical care. In addition, contrary to traditional methods, such as XGboost or CatBoost that can present uncertainty measures (e.g., 95% confidence interval), deep learning has greater difficulty in quantifying the uncertainty measures. In this paper, to describe the uncertainty information, we attempted to quantitatively measure the uncertainty as much as possible through bootstrapping [23]. In fact, recent attempts have been made to explain deep learning and measuring uncertainty, which will be our next area of study [36, 37]. Second, as this study was conducted in only two hospitals in Korea, it is necessary to validate the model for patients in EMSs in greater populations or other countries.

We developed a high-performance algorithm by combining an AI algorithm and a conventional triage tool. Despite several limitations, deep learning achieved a high predictive performance in several medical domains. Further, the deep learning algorithm can be developed more easily than a machine learning method. Based on our methodologies and results, other researchers can develop algorithms for their own groups of patients and situations. Additionally, medical researchers could investigate the applicability and future development of deep learning in various domains of medicine. For example, using this algorithm, the need for the critical care of patients could be predicted during EMS situations, and the destination hospital could be optimized by considering the predicted critical care needs and hospital’s situation (e.g., ED overcrowding, ICU capacity, and critical care availability). Moreover, the predictor variables in this algorithm were simple and could be used via a wearable device and information from a patient or their family. Because of this, patients with severe underlying diseases could be monitored daily while they are living at home regarding their needs for critical care, and could be referred to hospital earlier if they exhibit deterioration.

## Conclusion

In this study, a triage using an AI algorithm accurately predicted the need for critical care of patients using information during EMS situations, and outperformed the conventional triage tools and early warning scores. The results showed the potential of AI for EMSs, which will be a useful and fast tool to identify vulnerable patients and help precise decision-making in daily practice.

## Supplementary information

**Additional file 1.** Supplemental material. Brier scores and calibration plot.

## Data Availability

The data underlying this study belong to the National Emergency Medical Center (NEMC) of Korea. NEMC provides de-identified national emergency department information system data to researchers for nonprofit academic research. Any researchers who propose a study subject and plans with a standardized proposal form, and are approved by the NEMC review committee on research support can access the raw data. Details of this process and a provision guide are now available at the NEMC website (https://dw.nemc.or.kr) or contact point of the NEMC review committee (skko@nmc.or.kr). The authors accessed the data used in this study in the same manner that they expect future researchers to do so and did not receive special privileges from the NEMC of Korea.

## Abbreviations

AI: Artificial intelligence
AUC: Area under the receiver operating characteristic curve
DNN: Deep neural network
ED: Emergency department
EMS: Emergency medical service
ESI: Emergency Severity Index
ICU: Intensive care unit
KTAS: Korean triage and acuity system
MEWS: Modified early warning score
NEDIS: National emergency department information system
NEWS: National early warning score
NPV: Negative predictive value
PPV: Positive predictive value
ReLU: Rectified linear unit

## References

[1] Moore L (1999). Measuring quality and effectiveness of prehospital ems. Prehospital Emerg Care.

[2] Baxt WG, Jones G, Fortlage D (1990). The trauma triage rule: a new, resource-based approach to the prehospital identification of major trauma victims. Ann Emerg Med.

[3] Lidal IB, Holte HH, Vist GE (2013). Triage systems for pre-hospital emergency medical services - a systematic review. Scand J Trauma Resusc Emerg Med.

[4] Seymour CW (2010). Prediction of critical illness during out-of-hospital emergency care. JAMA.

[5] Kahn JM, Branas CC, Schwab CW, Asch DA (2008). Regionalization of medical critical care: what can we learn from the trauma experience?. Crit Care Med.

[6] Evans SM, Murray A, Patrick I, Fitzgerald M, Smith S, Andrianopoulos N (2010). Assessing clinical handover between paramedics and the trauma team. Injury.

[7] Dojmi Di Delupis F, Pisanelli P, Di Luccio G, Kennedy M, Tellini S, Nenci N (2014). Communication during handover in the pre-hospital/hospital interface in Italy: from evaluation to implementation of multidisciplinary training through high-fidelity simulation. Intern Emerg Med.

[8] Gulshan V, Peng L, Coram M, Stumpe MC, Wu D, Narayanaswamy A (2016). Development and validation of a deep learning algorithm for detection of diabetic retinopathy in retinal fundus photographs. Jama.

[9] Kwon J, Jeon K-H, Kim HM, Kim MJ, Lim S, Kim K-H (2019). Deep-learning-based out-of-hospital cardiac arrest prognostic system to predict clinical outcomes. Resuscitation.

[10] Breiman L. Statistical Modeling : The Two Cultures. 2011;16:199–215.

[11] Kwon Joon-myoung, Lee Youngnam, Lee Yeha, Lee Seungwoo, Park Hyunho, Park Jinsik (2018). Validation of deep-learning-based triage and acuity score using a large national dataset. PLOS ONE.

[12] Schalkoff RJ (1992). Pattern recognition - statistical, structural and neural approaches.

[13] Ioffe S, Szegedy C (2015). Batch Normalization: Accelerating Deep Network Training by Reducing Internal Covariate Shift.

[14] Abadi M, Barham P, Chen J, Chen Z, Davis A, Dean J (2016). TensorFlow: A System for Large-Scale Machine Learning TensorFlow: A system for large-scale machine learning. 12th USENIX Symp Oper Syst Des Implement (OSDI ‘16).

[15] Gilboy N, Tanabe P, Travers D (2012). Rosenau AM. Emergency Severity Index (ESI): A Triage Tool for Emergency Department Care, Version 4: Implementation Handbook 2012 Edition.

[16] Mistry B, Stewart De Ramirez S, Kelen G, PSK S, Balhara KS, Levin S (2018). Accuracy and Reliability of Emergency Department Triage Using the Emergency Severity Index: An International Multicenter Assessment. Ann Emerg Med.

[17] Kwon H, Kim YJ, Jo YH, Lee JH, Lee JH, Kim J, et al. The Korean triage and acuity scale: associations with admission, disposition, mortality and length of stay in the emergency department. Int J Qual Heal Care. 2018. 10.1093/intqhc/mzy184 30165654

[18] Lee B, Kim DK, Park JD, Kwak YH (2017). Clinical considerations when applying vital signs in pediatric Korean triage and acuity scale. J Korean Med Sci.

[19] Subbe CP, Kruger M, Rutherford P, Gemmel L (2001). Validation of a modified early warning score in medical admissions. QJM.

[20] Smith GB, Prytherch DR, Meredith P, Schmidt PE, Featherstone PI (2013). The ability of the National Early Warning Score (NEWS) to discriminate patients at risk of early cardiac arrest, unanticipated intensive care unit admission, and death. Resuscitation.

[21] Williams TA, Tohira H, Finn J, Perkins GD, Ho KM (2016). The ability of early warning scores (EWS) to detect critical illness in the prehospital setting: a systematic review. Resuscitation.

[22] Jouffroy R, Saade A, Ellouze S, Carpentier A, Michaloux M, Carli P (2018). Prehospital triage of septic patients at the SAMU regulation: comparison of qSOFA, MRST, MEWS and PRESEP scores. Am J Emerg Med.

[23] Carpenter J, Bithell J (2000). Bootstrap confidence intervals: when, which, what? A practical guide for medical statisticians. Stat Med.

[24] Dietterich TG (2007). Ensemble methods in machine learning.

[25] Shu Eileen, Ives Tallman Crystal, Frye William, Boyajian Jonathan G., Farshidpour Leyla, Young Megann, Campagne Danielle (2019). Pre-hospital qSOFA as a predictor of sepsis and mortality. The American Journal of Emergency Medicine.

[26] Buschhorn HM, Strout TD, Sholl JM, Baumann MR (2013). Emergency medical services triage using the emergency severity index: is it reliable and valid?. J Emerg Nurs.

[27] Leeies M, Ffrench C, Strome T, Weldon E, Bullard M, Grierson R (2017). Prehospital application of the Canadian triage and acuity scale by emergency medical services. CJEM.

[28] Sun Guo-Wen, Shook Thomas L., Kay Gregory L. (1996). Inappropriate use of bivariable analysis to screen risk factors for use in multivariable analysis. Journal of Clinical Epidemiology.

[29] Bagley SC, White H, Golomb BA (2001). Logistic regression in the medical literature: standards for use and reporting, with particular attention to one medical domain. J Clin Epidemiol.

[30] LeCun Y, Bengio Y, Hinton G (2015). Deep learning. Nature.

[31] Kwon J, Lee Y, Lee Y, Lee S, Park J (2018). An algorithm based on deep learning for predicting in-hospital cardiac arrest. J Am Heart Assoc.

[32] Wolpert DH, Macready WG (1997). No free lunch theorems for optimization. IEEE Trans Evol Comput.

[33] Christ M, Grossmann F, Winter D, Bingisser R, Platz E (2010). Modern triage in the emergency department. Dtsch Arztebl Int.

[34] Dugas AF, Kirsch TD, Toerper M, Korley F, Yenokyan G, France D (2016). An electronic emergency triage system to improve patient distribution by critical outcomes. J Emerg Med.

[35] Checucci E, Autorino R, Cacciamani GE, Amparore D, De Cillis S, Piana A, et al. Artificial intelligence and neural networks in urology: current clinical applications. Minerva Urol Nefrol. 2019. 10.23736/S0393-2249.19.03613-0. 10.23736/S0393-2249.19.03613-0 31833725

[36] Chen X, Duan Y, Houthooft R, Schulman J, Sutskever I, Abbeel P (2016). InfoGAN: Interpretable Representation Learning by Information Maximizing Generative Adversarial Nets.

[37] Fong RC, Vedaldi A (2017). Interpretable Explanations of Black Boxes by Meaningful Perturbation. Proc IEEE Int Conf Comput Vis 2017.

