# Supplementary figures and images for: Artificial intelligence algorithm to predict the need for critical care in prehospital emergency medical services
Source: Scand J Trauma Resusc Emerg Med. 2020 Mar 4;28:17. doi: 10.1186/s13049-020-0713-4 (PMC7057604; doi:10.1186/s13049-020-0713-4)

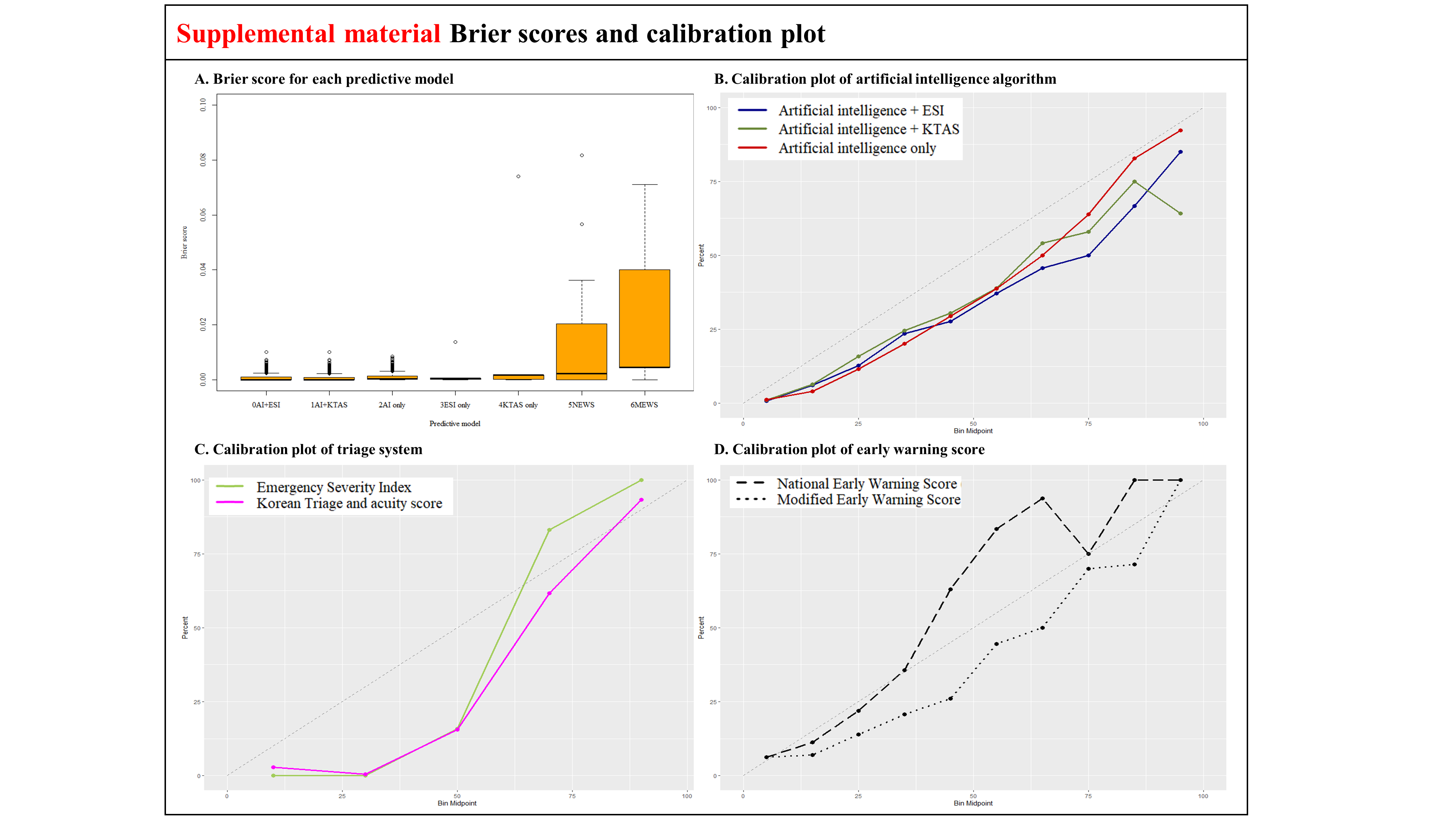

Supplement: Supplementary file 1 — Additional file 1. Supplemental material. Brier scores and calibration plot. [file 13049_2020_713_MOESM1_ESM.tif]
